# Caregiver Contribution to Heart Failure Self‐Care (CACHS)

**DOI:** 10.1002/nop2.35

**Published:** 2015-10-13

**Authors:** Karen Harkness, Harleah G. Buck, Heather Arthur, Sandra Carroll, Tammy Cosman, Michael McGillion, Sharon Kaasalainen, Jennifer Kryworuchko, Sheila O'Keefe‐McCarthy, Diana Sherifali, Patricia H. Strachan

**Affiliations:** ^1^ School of NursingMcMaster UniversityHamiltonONCanada; ^2^ Hamilton Health SciencesHamiltonONCanada; ^3^ Schreyer Honors CollegeHartford Center of Geriatric Nursing ExcellenceCollege of NursingThe Pennsylvania State UniversityPAUSA; ^4^ McMaster UniversityHamiltonONCanada; ^5^ Population Health Research InstituteHamiltonONCanada; ^6^ Cardiovascular Nursing ResearchHamiltonONCanada; ^7^ University of Saskatchewan College of NursingSaskatoonSKCanada; ^8^ Canadian Council of Cardiovascular Nurses/Ross Memorial HospitalLindsayONCanada; ^9^ Diabetes Care and Research ProgramHamilton Health SciencesHamiltonONCanada

**Keywords:** Caregiving, heart failure, instrument development

## Abstract

**Aim:**

While caregivers (CGs) make an important contribution to the self‐care of heart failure (HF) patients, there are no reliable and valid tools for measuring such contributions. Current interventions that strive to optimize patient outcomes through self‐care strategies neglect to account for CG contributions, a potential confounder on outcomes. The aim of the study was to develop an instrument that measures CG contributions to HF patients’ self‐care.

**Design:**

The study design follows an established process for instrument development.

**Methods:**

A systematic literature review and semi‐structured interviews of CGs were conducted to identify measureable CG activities. Items were derived from thematic analysis of CG narratives. A content validity index was computed for each item (I‐CVI). Items with an I‐CVI of >0·70 were retained. Items with an I‐CVI of 0·50–0·70 were revised for clarification and items with an I‐CVI <0·5 were discarded, except in instances where fulsome theoretical or empirical evidence supported their retention.

**Results:**

14 CGs completed interviews and 10 CGs with 4 expert nurses completed I‐CVI testing. Major interview themes included arranging appointments, medication adherence, monitoring, coordinating care, encouraging independence and taking action. A total of 36 items were constructed and underwent I‐CVI testing. Following I‐CVI testing, 27 items were retained, seven items were retained after revision based on CG feedback and two items were removed. This newly developed 34‐item questionnaire represents current literature, CGs’ experiences, excellent I‐CVI scores and ready for further psychometric testing.

## Introduction

### Background

Heart failure (HF) is a complex chronic syndrome associated with high mortality rates and frequent hospitalizations. In the United States, HF hospitalizations increased from 1·3 million in 1979‐3·9 million in 2004 (Fang *et al*. 2008). It is estimated that the prevalence of HF in the United States is expected to increase from approximately 5·8 million‐8·5 million in 2030 (Heidenreich *et al*. 2013). The Canadian Enhanced Feedback For Effective Cardiac Treatment phase 1 study (1991–2001; *n* = 8543) found that following hospital discharge for heart failure, the median survival time of HF patients was 1·75 years, with a 10‐year mortality rate of 98% (Chun *et al*. 2012). Worldwide, it is estimated that HF consumes between 1·1% and 1·9% of total healthcare expenditure in developed countries, with 50‐74% of the HF costs attributed to hospitalization or long term institutionalization (Liao *et al*. 2008).

Internationally, clinical practice guidelines recommend that promoting self‐care is fundamental to patient‐centred practice (Lindenfeld *et al*. 2010, McMurray *et al*. 2012, McKelvie *et al*. 2013). Engaging in self‐care is vital to symptom stability and improved health‐related quality of life (HRQL) (Buck *et al*. 2012, Lee *et al*. 2014). Optimally, comprehensive HF self‐care activities include: (a) managing multiple medications; (b) adhering to diet and fluid restrictions; (c) engaging in exercise; (d) monitoring symptoms and weight; (e) responding to changes in symptoms; and (f) navigating the health care system (Riegel *et al*. 2012).When HF patients do not consistently engage in self‐care activities, they are vulnerable to clinical deterioration, poor HRQL and frequent re‐hospitalization (Leventhal *et al*. 2005, Albert 2008, Riegel *et al*. 2009, Lee *et al*. 2011).

HF patients routinely depend on their informal caregivers (CGs), such as family members or friends, to help them with self‐care activities (Dickson *et al*. 2011, Davidson *et al*. 2013, Harkness *et al*. 2015). The CG contribution to HF patient self‐care has been described *indirectly* through reviews of the experience of CGs (Saunders 2008, Usher & Cammarata 2009, Kang *et al*. 2011) and family influences on HF patient self‐care (Clark *et al*. 2008, 2014, Dunbar *et al*. 2008, Gallagher *et al*. 2011, Oosterom‐Calo *et al*. 2012). Although the *direct* contributions of CG to HF patient outcomes have been explored broadly in several qualitative studies, they have yet to be categorized and examined quantitatively. Tools measuring the experience of CGs of patients with HF are available, but do not fully capture the activities to support patient self‐care described by CGs in the qualitative literature (Harkness & Tranmer 2007, Luttik *et al*. 2008). Recently, the Self‐care in Heart Failure Index (SCHFI), designed to measure patient engagement in self‐care, was modified to measure the caregiver contribution to self‐care (Vellone *et al*. 2013, 2014). However, the Caregiver Contribution to Self‐care in Heart Failure Index (CC‐SCHFI) may not capture the full CG experience (Vellone *et al*. 2013). For example, the CC‐SCHFI is completed by patients and reflects the patient's perception of the CG contribution rather than representing the actual voice of the CG. This is important for two reasons: the literature suggests that many CG contributions are ‘invisible’ and may go unnoticed by patients (Clark *et al*. 2008). Secondly, the CG's voice was not included in item development in the SCHFI (Riegel *et al*. 2000) and, therefore, modifying the SCHFI to capture CGs contributions to self‐care may not result in a comprehensive description or quantification of the actual CG‐specific contributions as identified by CGs. Therefore, an instrument with first person items, developed from and answered by CGs of patients with HF, is clearly needed. Valuable and valid information collected from such a questionnaire can then be used to guide strategies that help optimize patient and CGs’ collaborative approach to self‐care and ultimately minimize adverse clinical outcomes such as symptom deterioration, poor HRQOL and hospitalization.

## The study

### Aim

The purpose of this study was to develop an instrument to measure CG contributions to HF patients’ self‐care activities. Measurement of CG contributions to self‐care in HF patients will enable the further examination and quantification of: a) the degree of CG involvement in self‐care; b) the most common contributions made by CGs; c) the potential areas where CGs need assistance; and d) the impact of CGs contributions on patient outcomes.

### Methodology

**Figure 1:**
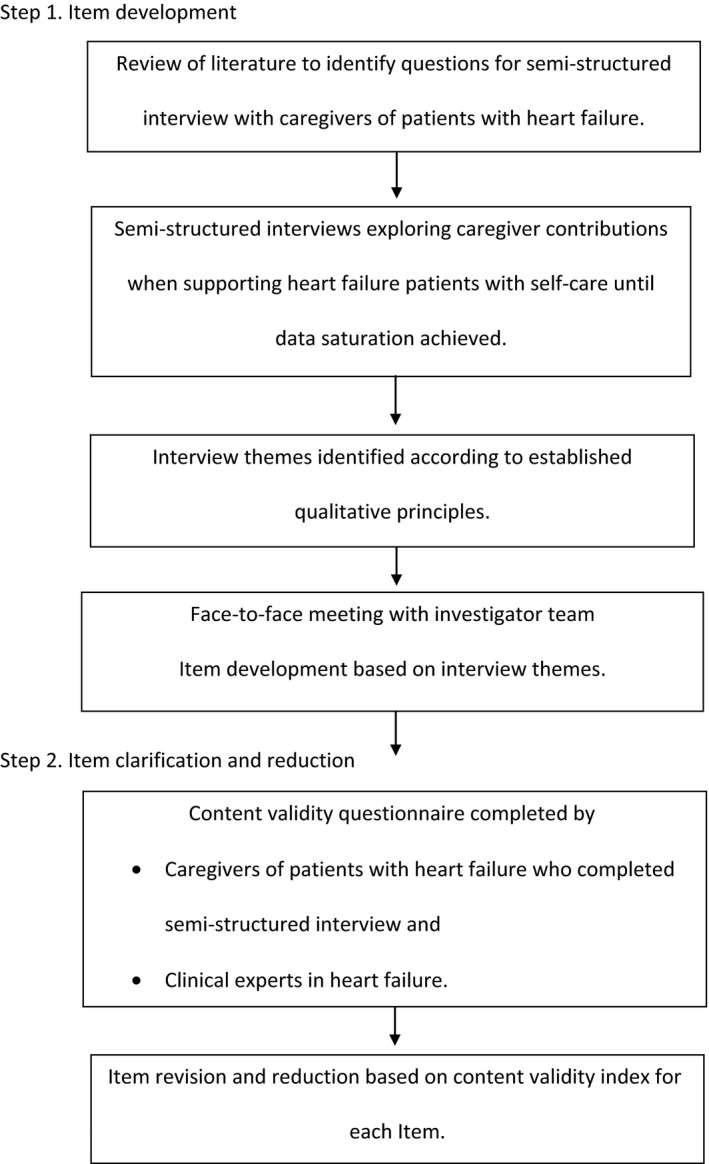
Study protocol.

The study design follows instrument development processes established by Kirshner and Guyatt (1985), Guyatt *et al*. (1992) and Juniper *et al*. (1994). Three distinct steps to the instrument development process include: Step 1. item development; Step 2. item clarification and reduction; and Step 3. initial psychometric testing. This study included Steps 1 and 2. Please see Figure 1 for an illustration of the protocol. The instrument developed and reported on in this study is known as The Caregiver Contribution to Heart Failure Self‐care (CACHS).

#### Step 1. Item Development

##### Review of the literature and interview guide development

We conducted a comprehensive, systematic review of the literature to identify measurable CG activities (Buck *et al*. 2015).Based on this work we developed a semi‐structured interview guide which was organized according to major theoretical sub‐domains from the Middle Range Theory of Self‐Care of Chronic Illness (Riegel *et al*. 2012). In this theory, self‐care is understood to include sub‐domains of: (1) maintenance (behaviours that maintain, preserve, or enhance health); (2) monitoring (cognitive processes and behaviours that result in vigilant body surveillance); and (3) management (attention to and then thoughtful response when bodily changes are noted during monitoring). These theoretical underpinnings guided item development processes.

##### Sample/participants

Inclusion criteria: We included adult (aged ≥18 years) informal CGs of patients (age ≥18 years) with a documented history of HF for at least 3 months. Exclusion criteria: CGs were excluded if they were: (1) caring for a patient who was pre‐ or postheart transplant or left ventricular device implantation; or (2) unable to read, write and understand English.

##### Study recruitment and ethical considerations

Eligible CGs were approached by health care providers during a routine patient follow up appointment in the outpatient Heart Function Clinic at the Hamilton Health Sciences. Interested CGs were then contacted by the research assistant and informed of the study objectives and procedures and asked for verbal consent to be contacted for an interview. Prior to the interview, eligible potential participants reviewed study information and provided informed consent. At the time of informed written consent, CGs were also asked if they were willing to be approached for Part 2 of the study. All CGs provided written consent for both Part 1 and Part 2 of the study. All nursing staff members working in the HF clinic at the time of the study were approached for inclusion and all provided consent. This study was approved by Hamilton Integrated Research Ethics Board (REB # 13‐041).

##### Caregiver interviews – data collection

Individual, face‐to‐face, semi‐structured interviews were subsequently conducted by an experienced interviewer to explore participants’ usual contributions to HF self‐care. Interviews were audio recorded and lasted between 30‐60 minutes. The digital audio‐recordings of the interviews were then transcribed, verbatim, by a professional transcriptionist; accuracy of each transcript was assessed by the interviewer. Our data collection and analysis process was iterative; completed participant interviews informed subsequent interview questions and probes. Following the initial four interviews, the principal investigators (KH, HB) analysed the transcripts for the depth and richness of the data to determine possible revisions to the interview guide. Initial interviews revealed that CGs had difficulty identifying specific self‐care tasks as these were part of their ‘usual routine’. To help with recall, the interviewer probed by giving examples of assistance identified by the literature and asked if this was similar to their routine. If so, CGs were asked to provide further explanation. However, CGs appeared to have ongoing difficulty thinking about assistance related to self‐management activities. Therefore, CGs were asked to describe a recent situation when the HF patient was not feeling well or required hospitalization. The interviewer was then able to ask for clarification and explanation about the process of symptom awareness and decision‐making during that specific event. After the first 6 interviews, no major changes to the interview guide were necessary.

##### Interview data analysis

Coding and thematic content analysis of the data was guided according to established approaches (Lynn 1986, Sandelowski 2000, Hsieh & Shannon 2005). Constant comparative analysis techniques were used to explore and understand the CGs activities (Strauss & Corbin 1998). The NViVO qualitative data management software program (International NVIVO 2012) was used to store and organize data analysis.

The interviewer and principal investigators (KH and HB) coded the first four interview transcripts independently and then met to reach agreement on a preliminary coding scheme. Initial coding categories emerged from these four interviews and all subsequent interviews were reviewed and grouped by the initial coding scheme categories. New categories that emerged from the data were added to the original coding scheme. Once the initial coding scheme was developed, further coding was completed by the interviewer and double coding by either KH or HB. The interviewer, KH and HB discussed the codes and grouped them into overall themes. Recruitment was discontinued when no further themes were identified by CG interviews and theoretical saturation from the initial literature search was achieved.

In a face‐to‐face full day meeting, our entire investigator team reviewed the results of the interview data analysis to derive a list of potential instrument items, based on distillation of clear categories of CG contributions to HF patients’ self‐care (Lynn 1986). During this process, our aim was to assure low inference‐based depictions of CG contributions to HF patients’ self‐care, thereby remaining as close as possible to the ‘everyday’ language of our participants. Potential items were then categorized into self‐care maintenance, symptom monitoring, or self‐management to assure that each theoretical sub‐domain was addressed. Disagreements were discussed until consensus was achieved on all items to be included in our instrument and subsequently subjected to content validity assessment (Step 2).

#### Step 2. Item Clarification and Reduction

The content validity of the developed instrument was assessed using a content validity questionnaire (CVQ). Content validity is the degree to which items contained in the instrument address important aspects of the construct being measured (Polit & Beck 2006). Content validity assessment is a process wherein consensus is sought among experts about the overall content of the instrument and individual item levels (Polit *et al*. 2007).

The content validity of the instrument was assessed based on the content validity index procedure described by Lynn (1986). Included with the CVQ was a detailed letter of explanation about the evaluation requirements and how to rank the items contained in the CVQ. Each item was ranked using a 4‐point rating scale to evaluate the: (a) clarity and wording of each item; (b) whether the item captured the contributions provided by CGs accurately; and (c) areas for possible modification or improvements (Lynn 1986). Response choices included: very relevant and easy to understand (4 points); relevant, but needs minor revision (3 points); unable to assess relevancy without item revision (2 points); not relevant (1 point). In this case, both formal and informal CGs were invited to complete the CVQ. Formal CGs constituted expert nurses who provide care for patients in the HF clinic; informal GCs were those who completed the initial interviews to derive item content. The research assistant was available to clarify questions while participants completed the CVQ.

##### Overall content validity index

An overall content validity index (CVI) based on the proportion of responses that indicated items scored as 4/4 (‘very relevant’) or 3/4 (‘relevant but needs minor revision’) was calculated for the total scale. Subscale CVIs were also calculated for each cluster of items representing theoretical sub‐domains of self‐care maintenance, symptom monitoring and self‐care management. The minimum acceptable overall CVI is considered to range from 0·8‐0·9, indicating an acceptable level of inter‐rater agreement for item relevancy (Polit & Beck 2006, Polit *et al*. 2007).

##### Individual content validity index

A CVI for each item (I‐CVI) was calculated by determining the proportion of responses per item scored 4/4 (‘very relevant’). Items with an I‐CVI of >0·70 were retained. Items with an I‐CVI of 0·50‐0·70 were revised for clarification. Items with an I‐CVI <0·5 were discarded, except in instances where fulsome theoretical or empirical evidence supported their retention.

## Results

Between March 2013‐September 2013, 14 informal CGs completed the original semi‐structured interviews. Content validity questionnaires were subsequently administered to the informal and professional CGs, between January 2014‐February 2014 (Step 2 described above). Four informal CGs, who completed the initial interview, were unable to complete the CVQ due to scheduling conflicts. Therefore, 10 informal CGs completed the CVQ. All of the expert nurses from the HF clinic (*n* = 4) completed the CVQ.

### Study sample

Those informal CGs (*n* = 14) who completed the original semi‐structured interviews were primarily female (79%), spouses (64%), residing with the HF patient (71%) with a mean age of 66 (SD 10) years. Four caregivers were daughters of the HF patients while 1 caregiver was the HF patient's son. The majority of CGs were retired (86%) and 2 CGs were employed. The CGs contributed to the self‐care of older HF patients (mean age 77 years) who were primarily male (57%) with advanced HF symptoms (71% New York Heart Association Class III symptoms). The four CGs lost to follow up were two wives and two adult children (1 son, 1 daughter) of the HF patients. The HF clinical experience for the four expert nurses (two expert nurse clinicians and two advanced practice nurses) who completed the CVQ in Step 2 ranged from 5‐10 years.

### Step 1‐Item Development

CGs described several activities and behaviours that supported and/or assisted HF patients with many sub‐domains of self‐care. The major themes that emerged from the semi‐structured interviews included:

- Arranging appointments
- Medication adherence
- Monitoring
- Coordinating Care
- Encouraging Independence
- Taking Action

Major categories and sub‐categories in these themes were organized using the theoretical self‐care sub‐domains from the Middle Range Theory of Self‐Care of Chronic Illness to develop instrument items (Riegel *et al*. 2012). Major themes and sample quotes followed by examples of instrument items are outlined in Table 1.

**Table 1:** Major themes that emerged from the semi‐structured interviews and sample quotes

Themes	Quotes	Sample Item
Encouraging independence	That's her area and the commode is in the bedroom. So, at night she can get there and it's right at the edge of the bed and so she… at night she's safe to get to the commode by herself. (CG04) And I do take her shopping. I would start off and go around… She likes to do it on her own. And I want her to keep as independent as possible. So, she'll go off and do her little bit of shopping and I'll just come in and check on her every once in a while. And she'll have a lot of stuff in her cart that's not right, as far as salt intake. So, then I try to use the process of saying ‘Let's have a look at this and check the sodium in it.’ And ‘Do you think this is a good choice for you to make?’ (CG06)	I make sure that he/she does as much as he/she can do on his/her own.
Arranging appointments	[Name] gives me the appointment, I mark it all down on the book and… and I have a book by the computer. I keep everything down. Like, he's all set up for all next week for three days straight. I keep his appointments and stuff like that. (CG05) So, she may have to take DARTS to one and I'll meet her there. So, she does the DARTS arrangements and sometimes she gets confused about that. And then she'll let me know and then I'll just… I do a phone call just to make sure that it's the right times and dates and everything else for her. (CG06)	I keep records or notes related to managing his/her heart failure. I arrange for medical appointments. I take him/her to his/her medical appointments.
Medication adherence	Well, I arranged… He was having awful trouble remembering about taking his pills. So, I arranged with the pharmacist to do dosettes for him. So, I pick up dosettes for him every week, so that they're already there. …But I noticed that he was forgetting to take them or he was taking them out of order. So, I put them in the kitchen and now I make sure that I give him [the pills] the pills, yeah. He's getting a lot better right now so he's…he's pretty good about taking them but I still have to check and make sure that he has. (CG07) Therefore put all his pills neatly into the three times a day. I set that up each week and then I put the morning pills into a little egg cup and then I can see whether he's taken them or not with his breakfast, with his lunch, with his supper. So, that's how we handle pills because I discovered when we didn't… when our doctor had not diagnosed the problem I realized that for three months he'd been doing goodness knows what with his pills and I wasn't aware of it. (CG16)	I talk with the pharmacist or health care providers about the medications on his/her behalf. I take responsibility for making sure his/her pills are organized. I could check (or make sure) he/she takes medications as prescribed.
Monitoring	Well, I… I look at her overall, you know, changes from what is normal and what is not normal. (CG01) I can tell by the colour of his face if he's going to have a bad day. [okay] I can tell just by looking at his face. The colour is not right in his face when he's going to have a bad day. (CG13) ..we were watching feet and hands and different places for swelling… all the symptoms. But then she built up fluid in the stomach. So, we didn't think she was in congestive heart failure. And the hospital said yes, she was. And since then she hasn't had it repeat because… I mean she gets monitored here very well and she's very aware of it herself and we're always looking for it too. (CG14)	I watch for any changes in his/her breathing. I watch for any changes in his/her swelling. I watch for any changes in his/her general appearance.
Coordinating care	I think for me, as a caregiver, I need to be informed. I can't just do what she says… the doctor said. I need to know. … I need to know about this new medication she's on……I write all the information down and pass it on so we all are filled in on what's going on at the moment. (CG14) He was that bad. But now he's fine, no problem at all. And I was there every day to make sure everything was going all right and explained to him. If he had tests done I would tell him what the tests were about and… (CG08)	I gather information about his/her heart failure or the topics that are important for his/her health. I talk with members of his/her health care team on his/her behalf.
Taking action	When she calls and says she's not feeling good, we go through kind of a list of questions; not planned, just ‘Okay, well how bad is it? Did it just start? How long has it been starting? Do you have chest pain? What are you actually feeling like so we can get a better picture? Do I need to call a doctor? Do I need to have her push medic alert (CG04) Well, then she had a problem really bad one day so we went to the hospital. And when we got to the hospital they increased the water pill by one and she was fine after that. So, now if it's bad we'll increase the water pill one extra. And it's the water on the lungs that are causing the breathing eh? (CG11)	I talk with him/her to help figure out what he/she is feeling. I help decide if we need to call someone for help or advice.

A total of 36 items were constructed and then organized in each theoretical sub‐domain: self‐care maintenance (16 items), symptom monitoring (9 items) and self‐care management (11 items).

### Step 2‐ Item Clarification and Reduction

The CVI for the entire instrument (*n* = 36 items) was 0·88. The CVI for each self‐care theoretical sub‐domain was as follows: 0·88 (self‐care maintenance); 0·96 (symptom monitoring); and 0·96 (self‐care management). These data reflect a high inter‐rater agreement across items and strong indicator of robust construct validity (Streiner & Norman 2008).

The majority of items (*n* = 27) had an I‐CVI >0·70 and were retained (without revision) in the instrument. For a summary of item CVI calculations, see Table 2. Seven items had an I‐CVI between 0·50 and 0·70 and therefore were retained after clarification, as based on CG suggestions. Item 1·5 had a CVI score 0·64 and was removed as it was anticipated to significantly correlate with item 1·6, which had a CVI score of 0·79. Item 3·9 was found to be confusing and CGs were unable to provide suggestions for revision. Therefore, item 3·9 was removed. Although item 1·1 had a CVI score of 0·5, it was retained as it is the only item that represents assistance with basic activities of daily living.

**Table 2:** Content validity scores for instrument items

Item		Item Content Validity Index
Self‐care maintenance		
1·1	I help with activities such as bathing, foot care, toileting, or dressing.	0·50
1·2	I arrange for services for help at home.	0·64
1·3	I arrange for medical appointments.	1·00
1·4	I take him/her to his/her medical appointments.	0·93
1·5	I make decisions related to food choices.	0·64
1·6	I help him/her decide what food choices are low in salt.	0·79
1·7	I help him/her decide how much fluid to drink each day.	0·50
1·8	I prepare the meals.	0·43
1·9	I organize his/her medications	0·50
1·10	I talk with the pharmacist or health care providers about the medications on his/her behalf.	0·79
1·11	I double check he/she takes medications as prescribed.	0·79
1·12	I encourage him/her to get some exercise every day.	0·71
1·13	I gather information about his/her heart failure or topics important for his/her health.	0·79
1·14	I keep records or notes related to managing his/her heart failure.	0·86
1·15	I talk with members of his/her health care team on his/her behalf.	0·86
1·16	I make sure that he/she does as much as he/she can do on his/her own.	0·79
Self‐care monitoring		
2·1	I watch for any changes in his/her breathing.	1·00
2·2	I watch for any changes in his/her swelling.	1·00
2·3	I watch for any changes in his/her weight.	0·86
2·4	I watch for any changes in his/her energy level.	0·86
2·5	I watch for any changes in his/her sleeping pattern.	0·86
2·6	I watch for any changes in his/her skin colour.	0·71
2·7	I watch for any changes in his/her general appearance.	0·79
2·8	I watch for any changes in his/her usual routine.	0·79
2·9	I pay attention to his/her emotional state.	1·00
Self‐care management		
3·1	I talk with him/her to help figure out what he/she is feeling.	0·93
3·2	I talk with him/her to help them calm down or relax during times when he/she feeling anxious or during moments of panic.	0·64
3·3	I help decide if we need to call someone for help or advice.	0·93
3·4	I call a friend or family member for support or advice or reassurance.	0·71
3·5	I call to make an appointment with a nurse or doctor.	0·86
3·6	I call and talk with a nurse or doctor for advice.	0·71
3·7	I suggest that he/she looks unwell and needs a rest.	0·86
3·8	I remove him/her from a situation if it looks like he/she is getting too tired or overwhelmed (e.g. social events).	0·71
3·9	I am able to figure out if *his/her* actions helped to make him/her feel better.	0·57
3·10	I am able to figure out if *my* suggestions or actions helped to make him/her feel better.	0·79
3·11	If we try something that doesn't help, I will try something else.	0·71

## Discussion

The purpose of this study was to develop a questionnaire using established principles for instrument development. The Caregivers Contribution to Heart Failure Self‐care (CACHS) questionnaire contains items that represent current evidence and the experience of CGs. Acceptable CVI scores suggest that the CACHS questionnaire has the potential to quantify key CG contributions to HF patient self‐care. To our knowledge, this is the first questionnaire developed specifically for measuring CG contributions to HF patients’ self‐care that overcomes the limitations of existing tools which were either designed and validated to measure the CG experience or burden (Harkness & Tranmer 2007, Luttik *et al*. 2008, Buck *et al*. 2015) or adapted from a pre‐existing patient instrument (Vellone *et al*. 2013) but are being used to quantify these contributions.

### Limitations

Specific limitations should be kept in mind when evaluating the findings. While we sought to recruit the most diverse group of CGs for the study, our sample was primarily retired females spouses residing with a (male) patient. However, to help minimize this limitation, the systematic review of all of the CG literature for HF patients (Buck *et al*. 2015) was used to derive a wide range of activities in the initial interview guide. We also continued interviews until no new activities were identified and theoretical saturation from the literature review was achieved. We recommend that future studies do further psychometric work in other CG populations such as ethnic and culturally diverse CGs and patients, CGs who may not reside with the patient and in a sample of working and unemployed CGs. Recruitment strategies also need to target CGs of patients who do not attend a HF clinic.

### Implications for research

The development of the CACHS questionnaire further advances research involving informal CGs of HF patients by capturing CG self‐report of their perceived contributions to HF patient self‐care. The CACHS questionnaire allows quantification of specific activities that CGs contribute to patients’ self‐care and will allow the impact of those activities on patient outcomes to be examined. CG activities were previously confounding variables in self‐care studies, which could only measure patient clinical variables or CG burden or mood states (Buck *et al*. 2015). Measurement of CGs contributions will allow assessment of the degree to which CGs engage in supporting HF patient self‐care when serving as co‐providers of care with clinicians. Furthermore, measuring CG contributions will help us to determine what degree of variability in patient self‐care is predicted by their CGs contribution. Finally, the CACHS questionnaire will also allow for more precise economic analysis.

### Implications for clinical practice

With further psychometric testing and refinement, the CACHS questionnaire may equip clinicians to assess and quantify the overall impact of the CGs on patient self‐care and target specific self‐care decisions or behaviours. For example, if the CG indicates on the questionnaire that he/she takes responsibility for the patient's medications, it may be more efficient and efficacious to target interventions to the CG who oversees organizing and administering HF medications. Thus, clinicians can ensure self‐care interventions are designed and supported to meet the contextual factors influencing self‐care and meet CG needs (Strachan *et al*. 2014). Current clinical guidelines recommend that patient (including families) centred care be evidence based. The CACHS questionnaire will contribute to building evidence for including CGs in HF patient education and decision‐making. Including CGs as partners is in keeping with nursing's holistic, lifespan models of care. Data accrued from studies using the CACHS questionnaire can be included in evidence based protocols and future quality improvement guidelines assuring that the very real and instrumental contributions of CGs are acknowledged and mobilized to improve HF patient outcomes.

## Conclusion

In this study, we reported on the development process and derivation of the content validity of the CACHS questionnaire to measure CGs contributions to HF patients’ self‐care. Thirty‐four items contained in the CACHS questionnaire met the CVI standards. The CACHS questionnaire should undergo validation in larger studies involving diverse populations to assess whether it remains to demonstrate robust and stable psychometric properties. The process of content validation reported in this publication is the initial step in a rigorous process to provide evidence for the validity of the assessment of CGs contributions to HF patient self‐care. Further research will be needed to establish the full psychometric properties of the CACHS questionnaire such as: reliability, predictive validity, responsiveness, sensitivity and specificity, interpretability, acceptability and feasibility.

## Conflict of interest

No conflict of interest has been declared by the authors.

## Author contributions

All authors have agreed on the final version and meet at least one of the following criteria [recommended by the ICMJE (http://www.icmje.org/recommendations/)]:

- substantial contributions to conception and design, acquisition of data or analysis and interpretation of data;
- drafting the article or revising it critically for important intellectual content.
